# Sirtuins and Autophagy in Age-Associated Neurodegenerative Diseases: Lessons from the *C. elegans* Model

**DOI:** 10.3390/ijms222212263

**Published:** 2021-11-12

**Authors:** Anam Naseer, Snober Shabnam Mir, Krisztina Takacs-Vellai, Aamir Nazir

**Affiliations:** 1 Academy of Scientific and Innovative Research (AcSIR), Ghaziabad 201002, India; anambbk@gmail.com; 2 Division of Neuroscience and Ageing Biology, CSIR-Central Drug Research Institute, Lucknow 226031, India; 3 Department of Bioengineering, Integral University, Lucknow 226026, India; smir@iul.ac.in; 4 Department of Biological Anthropology, Eötvös Lorand University, 1053 Budapest, Hungary; takacsve@gmail.com

**Keywords:** sirtuins, autophagy, neurodegenerative diseases, *C. elegans*

## Abstract

Age-associated neurodegenerative diseases are known to have “impaired protein clearance” as one of the key features causing their onset and progression. Hence, homeostasis is the key to maintaining balance throughout the cellular system as an organism ages. Any imbalance in the protein clearance machinery is responsible for accumulation of unwanted proteins, leading to pathological consequences—manifesting in neurodegeneration and associated debilitating outcomes. Multiple processes are involved in regulating this phenomenon; however, failure to regulate the autophagic machinery is a critical process that hampers the protein clearing pathway, leading to neurodegeneration. Another important and widely known component that plays a role in modulating neurodegeneration is a class of proteins called sirtuins. These are class III histone deacetylases (HDACs) that are known to regulate various vital processes such as longevity, genomic stability, transcription and DNA repair. These enzymes are also known to modulate neurodegeneration in an autophagy-dependent manner. Considering its genetic relevance and ease of studying disease-related endpoints in neurodegeneration, the model system *Caenorhabditis elegans* has been successfully employed in deciphering various functional outcomes related to critical protein molecules, cell death pathways and their association with ageing. This review summarizes the vital role of sirtuins and autophagy in ageing and neurodegeneration, in particular highlighting the knowledge obtained using the *C. elegans* model system.

## 1. Introduction

The brain is the most complex and a very important part of the body that is responsible for the perfect co-ordination and functioning of other organs. The precise functioning of the brain is achieved with the help of a very intricate network of brain cells, known as neurons, that form complex connections amongst each other. A neuron consists of the cell body that gives way to branches known as dendrites, and axons which help in communicating electric impulses or signals. These impulses are transferred to the next neuron via messengers known as neurotransmitters, across the synapse. This highly co-ordinated system requires a piece of high throughput machinery working in the background. However, as the body ages, neural communication—its overall functioning and its metabolism—weakens, leaving the aged body vulnerable to many age-associated diseases. Additionally, it is known that autophagy, one of the major clearance mechanisms, has an important role in the regulation of lifespan and the ageing process. It has been well established that reductions in autophagy lead to acceleration of the ageing process [1,2,3,4], and thatenhancement of autophagy also leads to anti-ageing effects on the body [5]. Any defect or dysfunctioning in this system results in a variety of ailments, and neurodegenerative diseases are one of them. Furthermore, an important class of enzymes known as sirtuins have been well studied and found to have a neuroprotective function; these class III HDACs are involved in autophagy-mediated regulation of neurodegeneration. Research over the past few decades has shed light on fundamental mechanisms associated with age-related diseases; however, gaps exist in converting mechanistic understanding into translational applications. This makes it imperative to decipher such mechanistic interfaces that allow delving into the prospective aspects of application and translation. The subsequent sections discuss the recent progress in sirtuin-mediated autophagy and its role in age-associated neurodegenerative diseases [6], highlighting the knowledge base that has been generated—particularly from research conducted employing the *C. elegans* model system. 

## 2. Neurodegeneration

Neurodegenerative diseases, as the name suggests, occur when brain cells lose their functionality and ultimately die. Neurodegeneration is a progressive and irreversible loss of neurons, either in terms of their structure or function. It involves multifactorial age-associated damage to the nerve cells that leads to difficulty in performing routine tasks, and if left unnoticed, then the condition might become detrimental, and also fatal. In research conducted during past years, a lot of factors have been associated with neurodegeneration, such as the inheritance of mutated genes, environmental or epigenetic factors, defects in protein clearance machinery and ageing. Out of these, one of the major risk factors for most neurodegenerative diseases is “age”. Other reasons for build-up of plaques or aggregates that result in dysfunctioning of the brain include brain insulin resistance, which decreases the responsiveness of neurons towards insulin via IRS-1-PI3K-Akt signaling pathway [7]; overstimulation of glutamate receptors— specifically NMDA receptors—(termed glutamate excitotoxicity) [8]; mitochondrial dysfunction [9]; and genes associated with neuronal support cells such as glial cells, which have been identified as enhancing the risk of neurodegeneration—for example, the APOE4 gene (predominantly found in astrocytes) is linked with an increased chance of developing Alzheimer’s disease (AD) [10]. Two major age-associated neurodegenerative diseases, affecting millions of people worldwide are discussed further.

### 2.1. Alzheimer’s Disease

Alzheimer’s disease (AD) is a major irreversible neurological disorder that brings about gradual loss of cognition, leading to a decline in quality of life. Analysis of epidemiological data and an increasing disease burden within society has led to AD becoming a global health priority of the World Health Organization (WHO) [11]. It is the leading cause of dementia, which hampers the daily routine of the patient and impairs memory, leading to an increased dependence upon others. The WHO reports that in the year 2020, around 50 million people suffered from dementia worldwide, of which about 60% lived in financially critical countries [12]. The disease affects women at a higher rate than men, making them account for two-thirds of the AD patient load [13,14,15]. 

AD is a multifactorial disease which occurs due to the excessive build-up of beta-amyloid (Aβ) plaques extracellularly, and hyperphosphorylated tau protein forming neurofibrillary tangles (NFTs) inside the cell. Normally, the proteolytic cleavage of APP (amyloid precursor protein)—a membrane-embedded protein condensed in synapses—by the alpha-secretase enzyme results in the formation of Aβ protein, which gets cleared up by protein clearance machinery. However, in the case of the disease pathology, the most accepted hypothesis suggests that APP gets cleaved by beta and gamma-secretase enzymes, leading to the accumulation of pathologic forms of Aβ protein (Aβ-40 or Aβ-42) in synaptic connections. Additionally, due to defects in protein clearance system, the condition further causes the formation of plaques [11]. 

On the other hand, hyperphosphorylation of tau protein (a microtubule-associated protein), responsible for maintaining the internal microtubular structure of the brain cell, causes loss of its integrity and functionality—thus forming fibrillary tangles. The aggregation of both the proteins: Aβ and tau, occurs primarily in the cortical regions of the brain, such as the isocortex, entorhinal region and hippocampus. Due to the involvement of hippocampus, AD pathology mainly affects learning and memory, and if left unattended the condition worsens, even leading to death [11,16]. 

Apart from this, there is another hypothesis (i.e., the cholinergic hypothesis) which states that AD pathology might also occur due to a decrease in the production of acetylcholine, reductions in acetylcholinesterase activity or loss of cholinergic neurons [17]. Although a plethora of work has been done in order to understand the mechanistic aspects of the disease, a large area of drug discovery remains to pose a bigger challenge, as there are currently no effective drugs available that can cure the illness. For Alzheimer’s disease, drug development pipeline data for 2020 show that there are 121 potential drugs currently in clinical trials. Out of these, 12 compounds have emerged as cognition enrichment agents and 12 have been found to be protective in ameliorating neuropsychiatric and behavioural symptoms. The remaining 97 have been recognized as disease-modifying therapeutics (DMTs) [18]. Drugs that are still considered as having a balancing function and as providing some relief in AD disease pathology include memantine, donepezil, galantamine and rivastigmine [19].

### 2.2. Parkinson’s Disease

After AD, Parkinson’s disease (PD) is known to be the most frequently reported neurodegenerative disease. It typically affects movement and locomotion and later on affects cognition as well. It leads to stiffness, shaking, bradykinesia, rapid eye movement sleep issues and difficulties in walking and balancing. As the disease progresses, it causes behavioural changes, sleep disturbances, resting tremors, issues with posture and memory difficulties [20]. 

The crude count of disease incidence is around 4.5–19 per 100,000 in the population per year. Indian prevalence counts of PD, by 2016, have been thought to have reached around 575,946 [21]. Additionally, it affects 50% more men than women [20]. It occurs due to the progressive loss of dopaminergic neurons in the substantia nigra pars compacta region of the brain, resulting in a reduction in dopamine content, and causing the movement-related pathology. The non-movement symptoms can be attributed to the loss of another important chemical messenger, norepinephrine. Levels of norepinephrine decrease greatly, as compared to their normal concentrations, throughout the brain in cases of PD [22]. Additionally, one of the important mechanisms to PD pathology includes the formation of Lewy bodies containing aggregates of misfolded α-Synuclein protein, a highly conserved neuronal phosphoprotein (coded by SNCA gene), inside dopaminergic neurons [20]. 

A recent study has shown that upon deregulation, α-Synuclein protein droplets via an intermediate liquid–liquid phase separation irreversibly mature into gel-like structures that sequester other cellular components, leading to pathology.

Apart from α-Synuclein accumulation, Parkinson’s pathology can also be attributed to mutations in genes such as PINK1 (PTEN-induced putative kinase 1), DJ-1, PRKN (parkin) and LRRK2 (leucine-rich repeat kinase 2). Here, parkin is a E3 ubiquitin ligase protein that has a protective function against mutant α-Synuclein, while PINK1 and DJ-1 are required for the normal functioning of mitochondria [23]. 

As an evident fact, it is very well known that nature is an epitome of balance; every cellular process occurring inside an organism’s body is required to function in a regulated manner in order to maintain homeostasis. As a consequence of the aggregation of proteins in the neuronal circuit, cells have mechanisms that mediate the clearance of these aggregates. One such phenomenon is called autophagy, which forms a part of the protein quality-control machinery. As such, it is very important to know in detail how autophagy functions and how it is involved in regulating this balance.

## 3. Autophagy: Friend or Foe

Autophagy, the term coined by Christian De Duve in 1963, is a conserved intracytoplasmic protein degradation pathway. It is involved in the delivery of cytoplasmic contents to the lysosome for degradation and recycling via the formation of a double membrane-bound vesicle called the autophagosome. This was initially thought to be triggered by stressful conditions such as nutrient deprivation, hypoxia, pathogenic invasion, oxidative stress, etc. [24]; however, there are reports which suggest that autophagy can be induced under non-starved conditions [25] as well. 

Also regarded as self-eating, autophagy is considered to have a protective function in nature, which is very much evident in studies of various model organisms, such as *Caenorhabditis elegans* and *Drosophila,* where autophagy upregulation leads to increases in longevity via different mechanisms such as nutrient deprivation, alterations in mitosis and mitochondrial turnover [25].

This is a highly regulated process, whose overactivation or suppression can lead to many pathological conditions such as cancer, muscle disorders, psychiatric disorders, pathogenic attack and neurodegeneration [26,27]. However, a basal level of autophagy is required by the cells to function in a normal manner. Besides increasing stress resistance and longevity, it has also been reported that a certain amount of stress, such as high temperatures (hormetic heat stress), is beneficial for autophagy-dependent proteostasis [1,2]. Based on the type of target molecule and cellular environment, the mammalian system carries out three main types of autophagy, namely: microautophagy, macroautophagy and chaperone-mediated autophagy [28,29]. Out of these, macroautophagy is currently the most studied. 

Macroautophagy proceeds with the formation of a lipid bilayer autophagosome that engulfs and carries the cargo to lysosomes for degradation by lysosomal hydrolases. However, in microautophagy, autophagosome formation is not seen—instead, the target gets directly incorporated into the lysosome via invagination of the lysosomal membrane. In the third type, chaperone-mediated autophagy, the target protein gets marked by a specific pentapeptide sequence which is then recognized and transported via chaperones to the lysosomal membrane, and with the help of membrane-associated proteins such as LAMPs (lysosome-associated membrane proteins), it enters the lysosomal cytoplasm for degradation [29]. Inclusion of ubiquitinated cargo into the autophagosome for lysosomal degradation is mediated via autophagosome-associated protein light chain 3 (LC3) [25]. Recent studies have characterized selective autophagy on the basis of the cargo it degrades. According to this, the sub-categories of macroautophagy include: mitophagy, ribophagy, lipophagy and pexophagy, to name a few [30]. 

Over the past few years, research in the field of autophagy has expanded further. After the Nobel prize for the discovery of autophagy mechanism by Yoshinori Ohsumi (2016), scientists have linked autophagy with various other factors, such as ageing [31], neurodegeneration, metabolism regulation, etc. As mentioned earlier, being a protective mechanism, deregulation of autophagic machinery leads to the build-up of many disease-like conditions including neurodegenerative diseases such as Alzheimer’s and Parkinson’s disease. The role of autophagy in regulating neurodegenerative diseases has now become evident in many studies [29,32,33]. It has been shown that deletion of autophagy-related genes results in a variety of cancers such as prostate, breast and ovarian cancers [34]. 

The autophagy mechanism includes five basic steps: the initiation/nucleation phase that starts with the formation of a phagophore or isolation membrane, which extends from its edges during the elongation phase and, with the help of the PAS (the phagophore-associated site) machinery, matures to form the autophagosome (maturation phase); the fusion phase, where docking and fusion of the autophagosome with the lysosome occurs, encircling and transferring the material to be degraded to the lysosome, which then gets degraded with the help of lysosomal hydrolases during the degradation phase—the resulting products are recycled for utilization by the cell [29,35].

**Figure 1:**
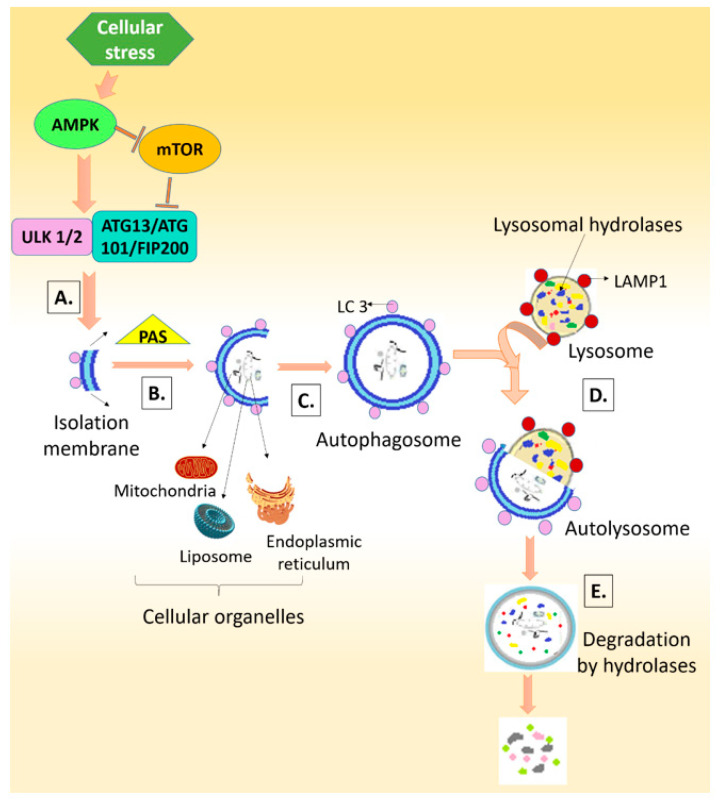
Process of autophagy. A. Initiation phase B. Elongation phase C. Maturation phase D. Docking and fusion phase E. Degradation phase.

Autophagy can also be induced by ROS, generated as a result of stressful conditions such as starvation or pathogenic attack. Nutrient deprivation in the cell, as marked by a higher AMP/ATP ratio, is sensed by mTORC1 (mammalian target of rapamycin complex 1) and AMPK (AMP-dependent protein kinase) which further regulate ULK1/2 (mammalian homologs of the *C. elegans* uncoordinated-51 kinase) to stimulate the formation of complexes containing ATG13, FIP200 (focal adhesion kinase family-interacting protein of 200 kDa) and ATG101, which further mediate autophagosome formation, leading to the initiation of autophagy [25] (Figure 1).

Although the genes required during the process of autophagy (ATG genes) were discovered in yeast (*Saccharomyces cerevisiae*), their importance in other model organisms is also enormous [29]. This attracts our attention to a very important model organism which is nowadays receiving much limelight in this field—that of *C. elegans*. After breakthrough discoveries such as apoptosis, RNA interference and the use of fluorescent proteins in a living system, *C. elegans* has become one of the most studied in vivo model systems for understanding disease physiology, finding therapeutic targets and studying many important mechanistic aspects of ageing, behaviour, development and neuronal health—to name a few. 

## 4. *C. elegans*: A Model for Research

*C. elegans,* a free-living soil nematode belonging to the Rhabditidae family, has gained the attention of many researchers since its recognition by Sydney Brenner in 1963 as a model organism. It is a small-sized unsegmented pseudo-coelomate; adults measure up to 1 mm with a transparent body, making them a wonderful model for studying anatomical aspects. Normally, it exists as a hermaphrodite, with males having a <0.2% chance of occurrence [36]. It is an easy-to-maintain model in the laboratory, as it is a bacterivore that feeds on *Escherichia coli*. 

One of the very important aspects of using this organism as a model system is that it has an almost 65% genetic homology with humans and that around 7500 *C. elegans* genes have human homologs [37]. It became the first multicellular animal to get its genome sequenced in 1998 by John Sulston and Robert Waterston. The lifespan of this organism is around 21 days (wild-type) and its generation time is ~3 days; this very feature makes it easy to observe desired outcomes throughout its lifespan within a very limited time. Additionally, an adult *C. elegans* worm lays around 300 eggs during its reproductive cycle; thus, a large number of progeny can be obtained with very few adult worms [38]. Another peculiar feature of *C. elegans* is that it has a fixed number of somatic cells, which have been mapped and studied. 

*C. elegans* offers a facile model for studying neurodegeneration. Of interest, in terms of extrapolating the findings from worms to humans, is the fact that many transgenic *C. elegans* strains expressing human proteins have been developed to study disease-associated pathologies. For example, in cases of PD and AD, there are strains which model the disease conditions such as α-Synuclein aggregation or Aβ aggregation, respectively; also, strains have been engineered with specific neurons tagged to fluorescent proteins that make it easier to track the effects of a particular condition on the degeneration of those specific neurons. One of these strains for PD includes NL5901, which has human α-Synuclein tagged with YFP and is expressed in muscles under the *unc-54* promotor; this strain has been widely used for the screening of compounds with therapeutic potential, such as *Bacopa monneri* extract [39]. Another strain, BZ555, expresses GFP under the influence of the dopamine transporter DAT-1, which makes it feasible to study dopaminergic neurons within this strain as the eight dopaminergic neurons of the worm exhibit fluorescence. This feature has been utilized in studies, where it was found that calorie restriction lowers dopaminergic neuronal loss via *sir-2.1* [40]. Apart from this, various models for AD have also been established, which include strain CL2006, which exhibits aggregation of Aβ under the control of constitutive the muscle-specific *unc-54* promotor—thus finding its place as a drug screening model for Aβ toxicity. Therapeutic compound-related studies that have used CL2006 for screening purposes include natural (extracts of plants such as *Gingko biloba* [41], soy isoflavone glycitein [42] and synthetic derivatives (thioflavin T [43], fluoxetine [44], reserpine [45]). Apart from developing transgenic strains, *C. elegans* can also be utilized in studies such as forward and reverse genetic approaches for better understanding of physiology and metabolism. One such approach is the use of the RNA interference technique (RNAi). Silencing of specific genes via RNAi has been well studied in *C. elegans* and this has also been successfully exploited in screening of potential therapeutic targets [46,47].

Another very important phenomenon, that is, autophagy, has been well-studied in this model [48,49,50]. Many orthologs of yeast ATG genes have been found in *C. elegans* that are involved in key steps such as let-363/TOR1, TOR2 and unc-51/ATG1—responsible for the induction of autophagy; bec-1/ATG6 and vps-34/VPS34—for autophagosome nucleation; atg-5/ATG5, atg-7/ATG7, lgg-1/ATG8 and lgg-3/ATG12—for protein conjugation; and atg-9/ATG9 and atg-18/ATG18—for retrieval and recycling [35,50]. A variety of strains have been established to study autophagy; for example, the DA2123 strain, which has GFP-tagged LGG-1 as an autophagy marker, which shows autophagosomes in the form of puncta within seam cells (a group of hypodermal cells arranged in longitudinal rows, lying along each side of the *C. elegans* body and undergoing multiple divisions during larval development [51,52] [4,53].

In *C. elegans,* many prime signaling mechanisms for autophagy regulation, as found in other eukaryotes, are conserved (Table 1). The key regulators of autophagy in *C. elegans* include serine/threonine kinase mTOR signaling [1] and Bafilomycin A [31] as inhibitors, and HLH-30 (an orthologue of the mammalian TFEB) and DAF-16 (an orthologue of the mammalian FOXO) as activators [54]. mTORC1 signaling downregulates the activity of the ULK1 complex, thus repressing autophagy induction. mTORC1 signaling is further regulated by nutrient-sensing kinases such as the AMPK and Akt pathways [55]. Additionally, PI3K acts as a major upstream modulator of mTOR and can further regulate autophagy via different stimuli such as growth factors or insulin [56]. In addition to these, bafilomycin A_1_ inhibits autophagosome-lysosome fusion by blocking the activity of the V-ATPase pump, thus inhibiting the autophagy process as a whole [57,58,59,60].

## 5. Sirtuins: Master Regulators

Neurodegeneration is a condition which results in the disruption of cellular homeostasis. This implicates a connection with one of the most important class of enzymes, sirtuins, which are involved in various vital processes. Sirtuins are members of class III NAD+ (nicotinamide adenine dinucleotide)-dependent deacetylases [67,68], which catalyze the deacetylation of N-acetyl lysine residues; however, in addition to deacetylation, they can also remove other moieties—thus, recent studies more correctly identify them as ‘NAD+-dependent deacylases’. They regulate important biological functions such as ageing, longevity, DNA repair, transcription, genome stability and metabolism. The first sirtuin to be discovered was Sir2 (silent information regulator 2) in yeast (*Saccharomyces cerevisiae*), by the geneticist Amar Klar in the 1970s. It was found to be an important component for transcriptional repression of silent mating-type loci: HML (homothallic mating left) and HMR (homothallic mating right). Later on, in the 1990s, the search for similar proteins in other model organisms began. However, the longevity-promoting effects of Sir2 in yeast were discovered by Leonard P. Guarante in 1991. These proteins are found to be evolutionarily conserved, ranging from bacteria to humans [69]. However, their number varies from as large as seven sirtuins in vertebrates to one in bacteria. This difference in number is further confirmed by phylogenetic analysis, which reveals that during the course of evolution, sirtuins have been lost selectively—as is found in plants, nematodes and insects. Additionally, this loss is thought to be compensated for by the functional redundancy offered by the remaining members of the sirtuin family [70].

In humans, the seven known members of the sirtuin family have different locations and varying enzymatic activities, as shown in Table 2.

In the case of *C. elegans,* sirtuins have been classified into four categories: *sir-2.1, sir-2.2, sir-2.3* and *sir-2.4*; out of these, *sir-2.1* shows maximum homology with yeast sirtuin—that is, Sir2. The subcellular localization of sirtuins found in *C. elegans* include: *sir-2.1* (SIRT1) in the nucleus and cytoplasm, *sir-2.2 and sir-2.3* (SIRT4) in the mitochondria and *sir-2.4* (SIRT6) in the nucleus [77,78]. *sir-2.1* shows 71.3% homology with human SIRT1 (the most studied family member) and is found to be located in neurons in the head, muscle, hypodermis and intestine. However, it is also known that in cases of food deprivation, *sir-2.1* gets re-located to the nuclei of intestine and muscle cells [79]. Like other members of the HDAC family, sirtuins have zinc in their structure; however, unlike others, it is not involved in catalytic activity—rather it forms a structural component of the molecule [80]. The larger domain of the sirtuin molecule is formed by a Rossmann-fold structure that acts as a signature for NAD+-binding proteins and a smaller Zn-binding domain. Two important studies that have played a pivotal role in turning the focus of research towards *C. elegans* sirtuins include one reporting the lifespan extension of yeast upon overexpression of Sir2, delaying replicative senescence, and another showing the NAD+-dependent deacetylase activity of SIRT1 in mammals [81]. In *C. elegans, sir-2.1* is a well-known regulator of longevity; however, it has many other functions apart from lifespan extension and delaying of ageing. Major longevity pathways regulated by *sir2.1* in *C. elegans* include daf-16/FOXO signaling, insulin/IGF-1 signaling (IIS) and the AMPK pathway. Calorie restriction, another important phenomenon that has been found to extend lifespan, has been performed in *C. elegans*. Calorie restriction, also termed as dietary restriction, is found to be modulated by *sir-2.1*. *C. elegans* offers a whole-organismal environment for studying calorie restriction in detail with the help of specific mutations, such as knocking out the *eat-2* gene, which disturbs pharyngeal pumping—thus automatically reducing bacterial uptake [82], or the induction of RNAi by the *drl-1* gene that encodes the dietary restriction-like condition—thus mimicking the conditions of calorie restriction [83]. Besides lifespan extension, *sir-2.1* is also involved in the nutrient sensing pathway, maintenance of genomic stability, adult neurogenesis, DNA repair, microglial activation, the insulin signaling pathway, apoptosis, autophagy and metabolism regulation [72].

Other members of the sirtuin family in *C. elegans,* such as sir-2.2 and sir-2.3, are categorized as mitochondrial sirtuins. These are associated with the regulation of mitochondrial genes such as *sod-3* (superoxide dismutase-3), *ctl-1/2* (catalases) and the oxidative stress response. These molecules are located close to each other on the X-chromosome and have conserved sequences, showing around 73.5% homology in their sequences—thus, they show overlaps in their functions as well; however, their mechanisms of action for oxidative stress regulation or lifespan regulation are different [84,85]. *sir-2.4* has been found to be involved in regulating the assembly of stress granules in *C. elegans,* thus acting as a chromatin modifier similar to SIRT6 in mammals [75,86]. Under normal conditions, *sir-2.4* is found to be located in the nucleus; however, under stressful conditions, it shuttles to the cytoplasm and modulates the number of P-granules or stress granules in mammals. Thus, *sir-2.4* is important for regulating stress resistance. During stress, such as heat shock, it promotes DAF-16 acetylation—thus promoting the nuclear recruitment of DAF-16, and thereby regulating the expression of age and stress-associated genes [87,88]. One of the very important functional aspects of sirtuins in neurodegeneration is discussed further.

## 6. Sirtuins and Neurodegeneration

With the advent of the discovery of sirtuins, a large area of metabolic and mechanistic understanding of neuronal function and communication has been opened. Sirtuins, especially SIRT1, have been widely studied in the context of the nervous system, with a special focus on neurodegeneration. A variety of evidence suggests that sirtuins, mainly SIRT1 and SIRT2, have varying roles in neurodegenerative diseases. Neuronal activation of sirtuins promotes a reduction in beta-amyloid toxicity and brings about neuroprotection, as in cases of Alzheimer’s disease. For example, SIRT1, by inhibiting nuclear factor kappa B and by upregulating PGC-1alpha, relieves beta-amyloid aggregation. SIRT3, via the activation of FOXO3, leads to induction clustering of p62—also a common component of Lewy bodies in Parkinson’s disease [33]—on ubiquitinated mitochondrial substrates, alleviating AD-associated toxicity [89].

Additionally, there are reports which suggest that overexpression of SIRT1 stimulates degradation of α-Synuclein with the involvement of molecular chaperones [77]. Similarly, in *C. elegans, sir-2.1* is found to decrease α-Synuclein aggregation in transgenic strains expressing YFP-tagged α-Synuclein in the muscles [90]. These diverse properties of sirtuins have led to the development of various molecules as activators, such as resveratrol, and inhibitors, such as nicotinamide and sirtinol, which has further resulted in their recognition as potential therapeutics for targeting neurodegenerative diseases.

## 7. Sirtuins and Regulation of Autophagy in Neurodegenerative Diseases

Neurons and neuronal system form a very intricate machinery that controls or governs several important physiological processes. Autophagy is a very important phenomenon that regulates neuronal homeostasis. Neurons consist of axons and highly branched dendritic connections. The autophagosomes in the neurons are formed at the axonal tip and mature upon moving in a retrograde manner along the axon [91]. However, any change or dysfunction of this system creates havoc inside the brain, which results in internal as well as external damage. One such situation is the accumulation of misfolded or unfolded protein aggregates inside or between brain cells [92], as is seen in cases of neurodegenerative diseases.

Additionally, there are reports which suggest that autophagy impairment has a significant role in aggravating PD pathology [25,30,93,94,95,96]. Studies have found that overexpression of genes, such as LRRK2, whose mutation is associated with PD, result in increased levels of autophagy via the Ca^2+^-associated pathway. Additionally, DJ-1, responsible for protecting dopaminergic neurons against oxidative stress or mitochondrial defects, is one of the regulators of autophagy, whose loss-of-function mutation results in Parkinson’s disease [93].

Additionally, due to dysfunctioning of the autophagic machinery, clearance of misformed protein aggregates such as α-Synuclein in the case of Parkinson’s disease is hampered; this results in impaired neuronal communication and signaling. Thus, it has been suggested by many authors that autophagy upregulation, either by mTOR-dependent or mTOR-independent pathways, can be a potential therapeutic strategy for alleviating Parkinson’s disease [25,30].

There are studies which suggest that members of the sirtuin family modify different processes in an autophagy-dependent manner. For example, SIRT3 could perform a dual function of activating and inhibiting autophagy via activation of different signaling mechanisms, such as the Sirt3–AMPK pathway, the Sirt3–Foxo3a pathway, and the Sirt3–SOD–mitochondrial ROS pathway [97]. SIRT5 is involved in the regulation of mitophagy (clearance of damaged or impaired mitochondria via autophagy machinery) and mitochondrial dynamics [98]. SIRT1, one of the most studied sirtuins, downregulates many transcriptional factors such as p53, nuclear factor kappa B [99], peroxisome proliferator-activated receptor γ (PPARγ), PPARγ coactivator-1α (PGC-1α) [89], liver X receptor, and forkhead box O (FoxO), which all have neuroprotective functions. Levels of SIRT1 also depend upon the availability of food, where calorie restriction promotes the expression of SIRT1, which deacetylates and suppresses p53 activity (one of its downstream targets) and in-turn induces autophagy; however, p53 performs both functions—that is, nuclear p53 acts as an inducer of autophagy while cytoplasmic p53 acts as an inhibitor of autophagy [99]. A study has also shown that SIRT1 knockout or knockdown decreased the induction of autophagy even via resveratrol (sirtuin activator) treatment in human cells or via dietary restriction in *C. elegans*. Furthermore, in the case of Parkinson’s disease, sirtuin-induced autophagy leads to degradation of α-Synuclein aggregates. SIRT1 further acts as a negative regulator of the mTOR signaling pathway, which in turn negatively regulates autophagy—thus leading to the survival of neurons in the brain [99]. 

**Figure 2:**
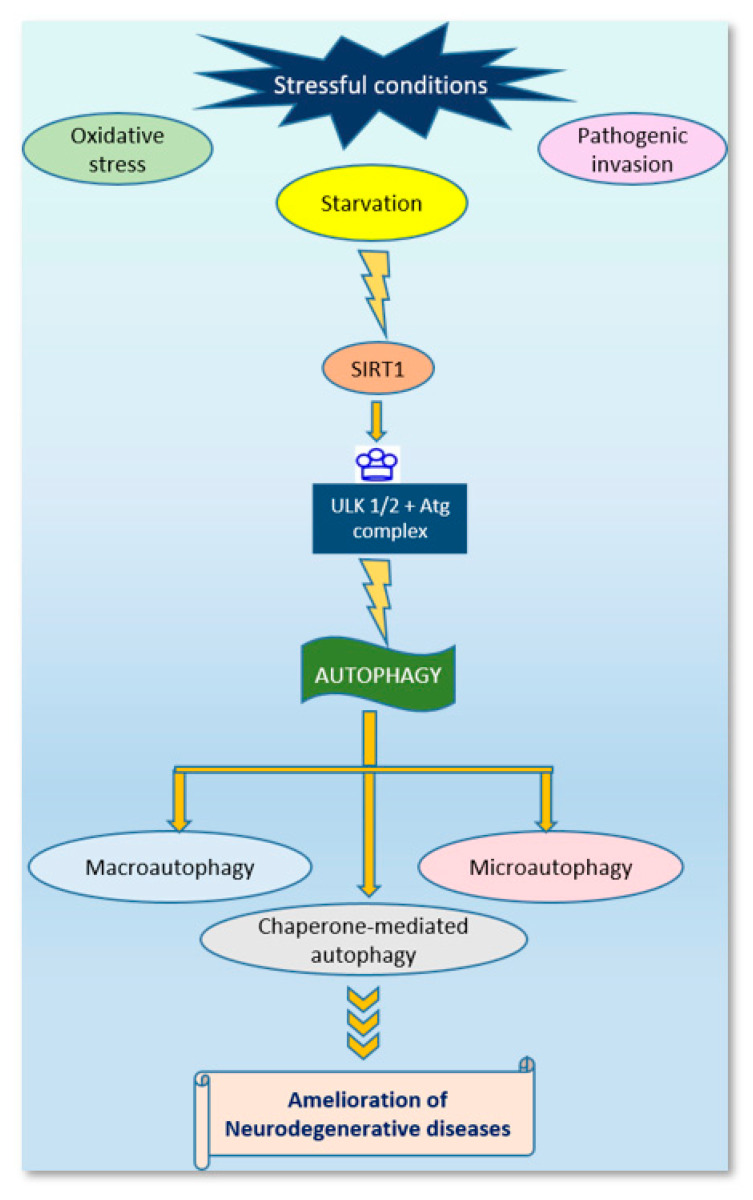
Schematic representation showing the involvement of SIRT1 in promoting autophagy and ameliorating neurodegeneration.

Moreover, SIRT1 forms complexes with autophagic components such as Atg7, Atg5 and LC3 and brings about their deacetylation [100]. Deacetylation of nuclear LC3 at K49 and K51 lysine residues promotes autophagy initiation under stressful conditions such as starvation [101] (Figure 2). Deficiency of sirtuin leads to decreases in the deacetylation of autophagy-related genes via the regulation of binding of transcription factors to promotors of autophagy genes [98]. Additionally, a proteasome activator, REG, inhibits SIRT1-mediated autophagy by degradation of SIRT1 in a ubiquitin-independent manner [102]. However, overexpression of sirtuin leads to the deacetylation of autophagy-related genes [98,103,104], which brings about the autophagy-dependent lysosomal degradation of target molecules or aggregated proteins or dysfunction of mitochondrial machinery— such as in the case of neurodegenerative diseases such as Alzheimer’s and Parkinson’s disease, respectively [105]. Studies suggest that under normal conditions, SIRT2 interacts with and deacetylates FOXO1 [106]; however, this association gets disturbed under oxidative stress, thus increasing the levels of acetylated FOXO1, which induces autophagy via its interaction with Atg7 [106]. Additionally, SIRT2 regulates mitophagy, the selective degradation of mitochondria [106]. Being a mitochondrial sirtuin, SIRT3 regulates the production of ATP, deacetylation of mitochondrial proteins, and mitophagy [107,108,109]. It also prevents glutamate excitotoxicity, thus preventing neurodegeneration [110,111]. SIRT3 protects cortical neurons during stress conditions by increasing the antioxidant capacity of mitochondria [110]. Thus, SIRT3 plays a very important role in regulating neurodegeneration in diseases such as Alzheimer’s and Parkinson’s disease [112,113,114,115,116]. Similarly, SIRT5 also protects against degeneration of dopaminergic neurons (hallmarks of PD) from MPTP (1-Methyl-4-phenyl-1,2,3,6-tetrahydropyridine: a selective neurotoxin which damages dopaminergic neurons in the nigrostriatal region of brain) by stimulating a higher antioxidant capacity in mitochondria [117]. SIRT6, mainly a nuclear sirtuin, induces autophagy and is thought to have a protective function [99]. SIRT6 and SIRT7 are known to repair DNA damage, and studies have shown that SIRT7 loss induces autophagy, which in turn regulates levels of TβRI (TGF-β receptor protein 1) protein, which further modulates TGF-β signaling—thus assisting in angiogenesis and wound healing [76,118].

Of note, during ageing, levels of sirtuins decrease, and there is also a reduction in autophagy. Thus, sirtuins and autophagy together play a very important role in regulating the ageing process. SIRT1 deficiency has been associated with hyperacetylation of phosphorylated tau, leading to reduced tau degradation via defective autophagy in cases of Alzheimer’s disease. Additionally, in *C. elegans, sir-2.1* SIRT1 homologue overexpression accelerates the formation of α-Synuclein aggregates in cases of Parkinson’s disease [28]. This fact is also supported by findings showing that knockdown or knockout of SIRT1 halts the induction of autophagy, even after treatment by resveratrol (an enhancer of sirtuin expression) in human cancer cells, or by calorie restriction in *C. elegans* [90,119].

## 8. Concluding Remarks

The regulation of metabolism and homeostasis is a prime necessity for the proper functioning of the body. This also means that there is always a need for fine-tuning between the production and consumption or degradation of materials generated during any process. Any disturbance in this pre-set regime creates a stressful environment in response to which the cell initiates certain activities to counteract its effects. One such activity is the activation of a class of deacetylating enzymes known as ‘sirtuins’, which further regulate multiple downstream targets. One of the vital phenomena with which sirtuins exert their function is via their role in ‘autophagy’. In recent years, a very important aspect of sirtuin-mediated autophagy in neuronal health has been a topic of interest. Neurons in the brain form a very intricate network. However, dysfunction of this system results in difficulties in performing routine tasks, leading to conditions such as neurodegeneration. Defects in sirtuin and autophagy machinery, either individually or in combination, result in the inter- or intra-neuronal accumulation of unfolded or misfolded proteins—leading to neurodegenerative diseases such as Alzheimer’s and Parkinson’s disease. To understand the mechanisms associated with sirtuin-mediated autophagic neurodegeneration, *C. elegans*, a model with immense genetic relevance, has proven to be a very useful model organism. It provides deep insights into the mechanistic and functional aspects of many cellular and physiological processes. However, constant research in this area has opened up a wide range of questions that are yet to be addressed, and researchers all around the globe are converging their endeavors in order to answer them and obtain a better understanding of critical aspects of ageing and neurodegeneration.

## Figures and Tables

**Table 1:** Regulatory molecules in mammals and *C. elegans,* governing the process of autophagy vis-a-vis the process of neurodegeneration.

S. No.	Pathways Regulating Autophagy	Regulatory Molecules	
		Mammals	*C. elegans*
1.	Nutrient/energy sensing pathway	mTORC1, Ras-cAMP-PKA, AMPK [61]	*let-363*, AMPK
2.	Insulin/growth factor signaling pathway	PKB/Akt, Ras-MAPK	DAF-2, Ras-MAPK
3.	Stress response pathway	IRE1α, PERK, ATF6α [62], ATG7, ATG8, SOD, CAT [63], HIF-1	*ire-1, pek-1, atf-6* [64], *atg7, lgg-1, sod-3, ctl-1/2*
4.	Pathogen-induced regulation	TLRs	TOL-1 [65]
5.	Transcriptional regulation and chromatin modification	FOXO, HDAC 1,2,3 (SIRT1-7) and 6 [66]	DAF-16, HDA-1 [66], *sir-2.1, sir-2.2, sir-2.3, sir-2.4*

**Table 2:** Human sirtuins and their *C. elegans* homologs; enzymatic activity and function.

S. No.	Human Sirtuin	Location	*C. elegans* Homolog	Enzymatic Activity	Function
1.	SIRT1	Nucleus (shuttles between nucleus and cytoplasm) [71]	*sir-2.1*	Deacetylase activity	Transcription regulation, cell survival chromatin organization, development and differentiation, stress responses, metabolism regulation, neuroprotection, adult neurogenesis, synaptic plasticity, cognition, emotion, circadian rhythm, microglial activation [72].
2.	SIRT2	Mainly cytoplasmic but can translocate to the nucleus as well		Deacetylase activity	DNA repair, cell cycle, mitosis, transcription regulation, adult neurogenesis, microglial activation, neuroprotection, regulation of emotions [72].
3.	SIRT3	Mitochondria [73]		Strong deacetylase activity	Mitochondrial functioning, metabolism regulation, ATP production, reducing oxidative stress, sleep-wake patterning, regulation of age-related hearing loss [72].
4.	SIRT4	Mitochondria	*sir-2.2* & *sir-2.3*	ADP-ribosyl transferase activity	Mitochondrial functioning, metabolism regulation
5.	SIRT5	Mitochondria		Desuccinylase and demalonylase activities; weak deacetylase activity	Fatty acid oxidation, insulin secretion [74]
6.	SIRT6	Nucleus (translocates to cytoplasm under stress) [75]	*sir-2.4*	Deacetylase activity, ADP-ribosyl transferase activity	Genome stability, DNA repair, control of circadian rythms, stress response, inflammation
7.	SIRT7	Nucleus, specifically in nucleolus		Deacetylase activity	Cell survival, rRNA regulation, cellular stress regulation [76]

## Abbreviations

HDAC	Histone Deacetylase
WHO	World Health Organization
Aβ	Beta-Amyloid
NFTs	Neurofibrillary Tangles
APP	Amyloid Precursor Protein
AD	Alzheimer’s Disease
DMTs	Disease Modifying Therapeutics
PD	Parkinson’s Disease
SNCA	Synuclein Alpha
PINK1	PTEN-induced Putative Kinase 1
PARKN	Parkin
DJ-1	Protein Deglycase
LRRK2	Leucine-Rich Repeat Kinase 2
LAMPs	Lysosome-Associated Membrane Proteins
LC3	Light Chain 3
PAS	Phagophore-Associated Site
YFP	Yellow fluorescent protein
GFP	Green fluorescent protein
LGG-1	LC3, GABARAP and GATE-16 family
AMP	Adenosine Monophosphate
ATP	Adenosine Triphosphate
AMPK	AMP-Activated Protein Kinase
mTORC	Mammalian Target of Rapamycin Complex
ULK1/2	UNC-51-like Autophagy-Activating Kinase 1/2
ATG	Autophagy-Related
RNA	Ribonucleic Acid
FOXO	Forkhead Box transcription factor
HLH	Helix-Loop-Helix
DAF-16	Abnormal DAuer Formation-16
TFEB	Transcription Factor EB
Akt	A Serine/Threonine Protein Kinase
PI3K	Phosphoinositide 3-Kinase
VATPase	Vacuolar ATPase
SIRT	Sirtuin
cAMP	Cyclic Adenosine Monophosphate
IRE1α	Inositol-requiring kinase 1α
PERK	Protein kinase (PKR)-like Endoplasmic
	Reticulum Kinase
ATF6	Activating Transcription Factor 6
CAT	Catalase
TLR	Toll-like Receptor
HIF-1	Hypoxia-Inducible Factor 1
NAD	Nicotinamide Adenine Dinucleotide
SOD	Superoxide Dismutase
ROS	Reactive Oxygen Species
PPARγ	Peroxisome Proliferator-Activated Receptor
	Gamma
PGC-1α	PPARγ Coactivator-1α
MPTP	1-Methyl-4-phenyl-1,2,3,6-tetrahydropyridine
TβRI	TGF-β Receptor Protein 1
